# The Influence of Upper and Lower Extremity Strength on Performance-Based Sarcopenia Assessment Tests

**DOI:** 10.3390/jfmk3040053

**Published:** 2018-11-03

**Authors:** Michael O. Harris-Love, Kimberly Benson, Erin Leasure, Bernadette Adams, Valerie McIntosh

**Affiliations:** 1 Muscle Morphology, Mechanics, and Performance Laboratory, Veterans Affairs Medical Center, Washington, DC 20422, USA; 2 Department of Exercise and Nutrition Sciences, The George Washington University Milken Institute School of Public Health, Washington, DC 20052, USA; 3 Geriatrics Service/Research Service, Veterans Affairs Medical Center, Washington, DC 20422, USA; 4 Physical Medicine and Rehabilitation Service, Veterans Affairs Medical Center, Washington, DC 20422, USA

**Keywords:** sarcopenia, strength assessment, lower extremity strength, hand grip strength, gait speed, physical performance, functional status

## Abstract

The optimal management of sarcopenia requires appropriate endpoint measures to determine intervention efficacy. While hand grip strength is a predictor of morbidity and mortality, lower extremity strength may be better associated with functional activities in comparison to hand grip strength. The purpose of our study was to examine the comparative association of upper and lower extremity strength with common measures of physical performance in older adults. Thirty community-dwelling men, aged 62.5 ± 9.2 years, completed body composition analysis, quantitative strength testing, and performance-based tests of functional status. Hand grip force values were not significantly associated with knee extensor or flexor torque values (*p* > 0.05). Hand grip force was only associated with fast gait speed, while knee extensor torque at 60°/s was the only variable significantly associated across all functional outcome measures: customary gait speed, fast gait speed, sit to stand time, and the Physical Performance Test (*p* < 0.02). Hand grip strength was not a proxy measure of lower extremity strength as assessed in this study. Overall, lower extremity muscle strength values had the strongest associations with participant functional performance. Lower extremity strength testing may provide additional value as an endpoint measure in the assessment and clinical management of sarcopenia.

## 1. Introduction

Sarcopenia is a condition that has been characterized by an overall loss in skeletal muscle mass with declines in strength or performance that progresses with age [1,2,3]. It is estimated that by age 70, individuals may incur a 40% loss of muscle mass and a concomitant 30% decrease in muscle strength [4]. This reduction of muscle mass and strength is associated with adverse health outcomes such as frailty, falls, decreased physical function, disability, and even death [2,4,5,6]. Older adults that are less physically active have been shown to be at greater risk for developing sarcopenia [7]. There is strong evidence in the literature that sarcopenia is a reversible cause of disablement, and that people in the early stages of sarcopenia would probably most benefit from forms of intervention [3]. Therefore, early detection coupled with activity-based interventions to promote adaptive muscle responses can help to combat the effects of sarcopenia [7,8,9]. Effective interventions for sarcopenia range from conventional weight training to alternative forms of exercise such as flywheel resistance training, strength training with intermittent vascular occlusion, exercise augmented with electrical stimulation, and whole-body vibration training [7,8,9]. The optimal management of geriatric syndromes depends on valid screening and diagnostic criteria, valid prevalence estimates, and appropriate endpoint measures to determine intervention effectiveness [3]. However, differing sarcopenia screening and staging criteria yield widely varying prevalence estimates and may result in undiagnosed cases for those in the early stages of sarcopenia. Consequently, the proportion of a patient cohort afforded intervention for sarcopenia is largely dependent on the selection of a given assessment tool or test criterion.

A general sarcopenia staging algorithm has been proposed by consensus groups such as the European Working Group on Sarcopenia in Older People (EWGSOP), and further refinements to the standard clinical assessment tools and classification criteria have been recently proposed by the Foundation for the National Institutes of Health (FNIH) and other investigative groups [2,10]. However, questions remain concerning commonly used standardized testing methods and screening criteria. The working definitions and prognostic measurements for sarcopenia have included combinations of muscle strength, gait speed, muscle mass, observed physical performance, and self-reported functional status [11,12]. Hand grip dynamometry has been often used to characterize muscle strength when assessing sarcopenia since hand grip strength is a strong predictor of disability, mortality, and overall muscle mass [3,13]. Due to the ease, reliability, and low cost of hand grip dynamometry, groups have also identified hand grip strength as an alternative initial screening method for sarcopenia in clinical settings [2,3,14]. However, researchers have also found that lower extremity strength may serve as a potential assessment measure for sarcopenia, since it is significantly associated with customary gait speed [15]. The decline in customary gait speed portends disability and has been linked with low muscle mass [3,14,16], although other factors such as visual acuity, balance, and joint integrity are important determinants of upright mobility status [17]. Given the minimal involvement of the finger and wrist flexors in gross mobility tasks, lower extremity strength may be better associated with functional activities in comparison to hand grip strength [2,18].

The purpose of this paper is to examine the comparative association of upper and lower extremity strength with common measures of physical performance in older adults. We hypothesized that knee extensor and flexor strength would have a stronger association with physical performance in comparison with grip strength. The overall goal of this work is to inform practitioners of the relative utility of upper and lower body strength testing in the identification and assessment of sarcopenia.

## 2. Materials and Methods

The participant data included in this report were obtained from the Age-Related Muscle Dysfunction Screening Study (ARMS Study I) [19], a prospective clinical trial that was conducted at the Washington DC Veterans Affairs Medical Center (DC VAMC). This study was approved by the DC VAMC Institutional Review Board and Research and Development Committee on 23 April, 2014, and registered with Clinicaltrials.gov (NCT02277236). Signed informed consent was obtained from all study participants prior to data collection.

### 2.1. Study Participants

The study participants were 30 community-dwelling male Veterans recruited from a Federal hospital as previously described [19]. Ten participants were classified as presarcopenic (<7.96 kg/m^2^) based on appendicular lean mass (aLM/ht^2^) cut off values from a large cohort study from a catchment area with patient demographics similar to our study site [20]. Ambulatory Veteran men between the ages of 45 and 85 receiving primary medical services at the DC VAMC were eligible for study participation (Table 1). Study candidates were excluded if they were recently hospitalized (within three months). Additionally, diabetes, edema, or musculoskeletal or neurological disorders that are associated with muscle atrophy precluded study participation. All participants within the sample of convenience completed the body composition analysis, strength testing, functional assessments, and anthropometric measures over the course of two visits within a range of 3 to 14 days. Body composition analysis was always performed on a separate visit from the physical performance tests.

### 2.2. Body Composition Analysis

Lean body mass (LBM) estimates via whole body DXA imaging using a GE Lunar iDXA machine were performed by a single trained DXA technician (GE Medical Systems Ultrasound and Primary Care Diagnostics, LLC, Madison, WI, USA). The summed regional estimates for the arms and legs (aLM) were calculated and scaled to height (aLM/ht^2^). All LBM estimates were calculated using the GE Encore v15 SP2 software package for the body composition data acquisition and analysis. Participant preparation and positioning for DXA was according to the GE DXA machine manufacturer’s manual and the DC VAMC Radiology Service testing procedures. Participant preparation for the DXA examination incorporated standardized instructions for maintaining normal hydration status, avoiding vigorous exercise prior to testing, and refraining from caffeine intake and food intake ≤ 6 h before the examination.

### 2.3. Quantitative Strength Testing

Hand grip dynamometry (Jamar, Lafayette Instruments, Lafayette, IN, USA) was used to assess upper extremity strength. Grip strength is a common measure of muscle function in older adults and a preferred method of strength assessment for sarcopenia clinical trials [11]. The participants observed a demonstration of the dynamometry test procedure and engaged in one to two practice attempts prior to data collection. The participants performed the test in the seated position with the shoulder adducted in alignment with the torso, elbow flexed at 90°, forearm and wrist in the neutral position, and proximal metacarpophalangeal joints flexed at approximately 90°. The duration of each trial was 5 s with 1 min of rest between trials. The mean value of three trials under standardized conditions were used to obtain the peak force values [21].

An isokinetic dynamometer was used to assess lower extremity strength (Biodex System 4, Biodex Medical Systems, Shirley, NY, USA). Knee extensor and flexor strength was assessed at isokinetic speeds of 60°/s and 180°/s using methods adapted from previously published protocols [22,23,24] with patient positioning and stabilization per the Biodex Operations Manual. A familiarization session was provided to the participants before data collection to orient each person to isokinetic testing. Participants were oriented to the visual feedback for the force-time curve displayed on the computer monitor, and provided with a familiarization session, prior to data collection. Net torque values were corrected for limb segment weight, and torque-time curves were assessed for movement artifacts and the attainment of the target angular velocity. The powerhead deceleration setting was adjusted to the lowest setting to allow for an optimal range of motion at the specified angular velocity. Repetitions were performed within a range of motion of 90° to 100° total excursion during the reciprocal isokinetic knee extension and flexion testing. Participants completed 4 to 6 repetitions of submaximal isokinetic knee extension and flexion at 180°/s as warm up activity before exercise testing. Approximately 2 min of rest was provided between the five-repetition testing bouts. Strength data were compiled from the mean value of the highest three peak torque values from a five-repetition test. All reported peak force and torque values were scaled to body weight.

### 2.4. Physical Performance Assessments

**Figure 1:**
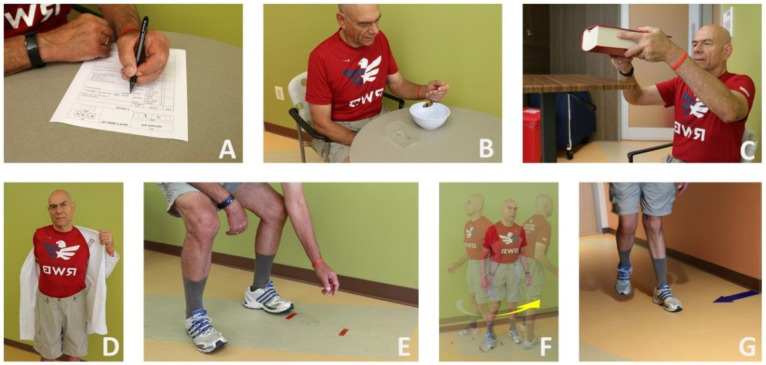
The 7-Item Physical Performance Test. The Physical Performance Test (PPT-7) is a performance-based functional assessment battery for older adults. The PPT-7 tasks include: (**A**) writing a sentence, (**B**) simulated eating, (**C**) lifting a book (approx. 3.2 kg) and placing it on a shelf (30.5 cm above shoulder level), (**D**) donning and doffing a jacket, (**E**) picking up a penny off the floor, (**F**) completing 360° turn, (**G**) and walking 15 m.

Physical performance was characterized using gait speed, the timed sit to stand test, and the Physical Performance Test (PPT-7). Gait speed is frequently used for sarcopenia screening and staging [11]. The gait speed test procedures used in this study were adapted from the 10-m gait speed as reported by Bohannon et al. and other investigators [25,26]. The cut-off value for slow gait speed was <1.0 m/s and has been used in other studies concerning sarcopenia screening [11]. A practice trial was permitted prior to data collection to assess participant safety and to confirm understanding of the test procedure. The customary gait speed test was completed prior to the fast gait speed test, and the lowest time of 2 trials was recorded for each testing condition. The timed sit to stand test has been used in sarcopenia studies to assess functional status or provide a clinical means to estimate muscle power [27,28]. The test movement was performed for five repetitions in a standardized manner as previously described [29]. Age-range specific cut-off scores were used to identify participants with impaired performance [30]. The functional battery used in this study was the modified version of the PPT-7. The PPT-7 was created as a performance-based assessment of functional assessment specifically for older adults (Figure 1) [31]. The individual activities that comprise the PPT-7 include: writing a sentence, simulated eating, turning 360°, putting on and removing a jacket, lifting a book and placing it on a shelf, picking up a penny from the floor, and walking approximately 15 m. The cut-off score for moderate frailty is a total score of <19.4 (scoring range, 0–28) and the test inter-rater reliability is suitable for clinical practice [32].

### 2.5. Statistical Analysis

Descriptive statistics were used to convey the outcome measures and participant characteristics with data expressed as means and standard deviations. Normality of the data distributions and variance distributions were assessed using the Shapiro-Wilk test and Levene’s test, respectively. Inferential statistics were used for an analysis of relationships among the sarcopenia assessment tests. Pearson product-moment correlation coefficients (*r*) were used to determine the association among the estimates of upper and lower extremity strength, and characterize the relationship of all strength measures with the performance-based functional outcomes. Partial correlations (*r*_xyz_) were used to further examine the association of strength with functional performance when adjusting for participant age [33]. The strength of the association among the correlation coefficients was described per the guidelines provided by Portney and Watkins [34]. Statistical analyses were performed using SPSS statistical software for Windows (version 10.0, SPSS Inc., Chicago, IL, USA). The α level was set at 0.05, and two-tailed *p* values < 0.05 were considered significant for all inferential statistics.

## 3. Results

The participants included 30 male Veterans who completed the study procedures without any complications or adverse events. The descriptive statistics for muscle strength and functional performance are provided in Table 2. Peak grip force values were not significantly associated with peak knee extensor torque (*r* = 0.28 and *r* = 0.06, *p* > 0.05) or peak knee flexor torque (*r* = 0.27 and *r* = 0.19, *p* > 0.05) across the 60°/s and 180°/s testing conditions, respectively. The gait performance of the study participants reflected customary gait speeds similar to age and gender-matched normative data, whereas the fast gait speed was approximately 17% slower (1.63 m/s) than published values [32]. The mean sit to stand time for the sample was 11.75 s ± 4.01 s, with eight participants exhibiting maneuver times below the estimated normative values for their respective age decade group [30]. The mean score for the PPT-7 was 21.2 ± 3.1 with the performance of the study participants attaining between the 50–75th percentile score based on normative data for older adults [31].

The Pearson correlations are reported in Table 3. There was a fair association between peak grip force and fast gait speed (*r* = 0.42, *p* = 0.02). However, peak grip force was not significantly associated with the other outcome measures. Peak knee extensor torque at 60°/s was the only strength variable to exhibit statistically significant correlations across all functional outcome measures: customary gait speed, fast gait speed, sit to stand, and PPT-7 (*p* < 0.02). Peak knee extension torque at 180°/s was significantly associated only with customary gait speed. In contrast, peak knee flexor torque at 60°/s and 180°/s exhibited statistically significant relationships with all outcome measures with the exception of the sit to stand test. The strongest relationships observed across all strength measures were for peak knee flexor torque at 180°/s with customary gait speed and the PPT-7.

**Figure 2:**
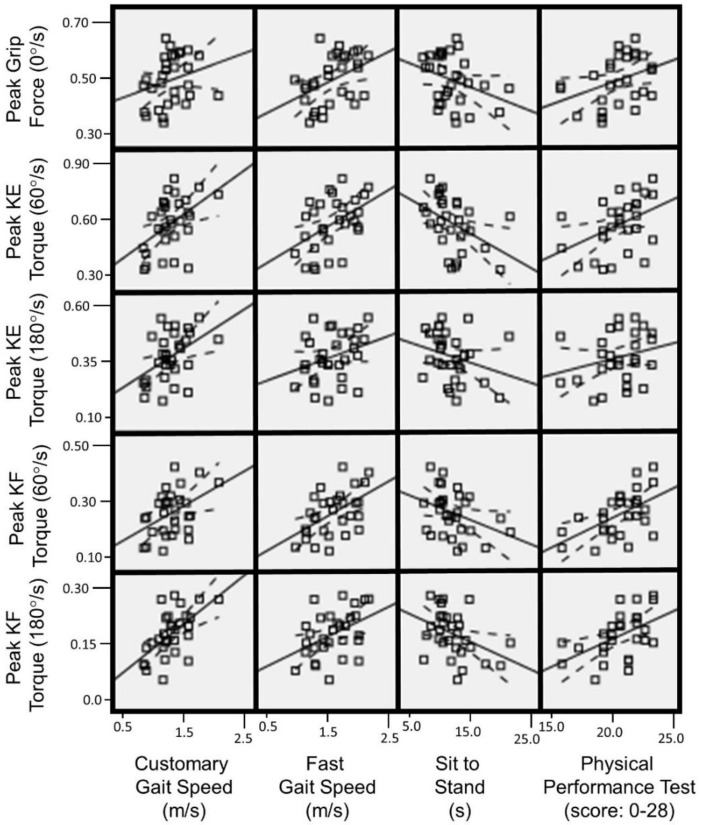
Matrix scatter plot for strength and physical performance measures (KE, knee extensor; KF, knee flexor; within each cell the solid line is the line of best fit, and the curved dashed lines are the 95% confidence intervals; strength values are scaled to body weight and are unitless; the Physical Performance Test is the seven-item version of the observed performance battery).

The age range within the study sample was 45 years to 83 years. Therefore, the observed strength-function relationships were further examined using partial correlations (*r*_xyz_) to adjust for the age of the participants. Significant relationships between peak grip force and fast gait speed (*r*_xyz_ = 0.37, *p* = 0.05), and peak knee flexor torque at 60°/s and customary gait speed (*r*_xyz_ = 0.37, *p* = 0.05), were not retained when adjusting for age. In addition, the associations between peak knee extensor torque and functional performance were not significant with partial correlations accounting for age. In contrast, peak knee flexor torque at 60°/s was significantly associated with fast gait speed (*r*_xyz_ = 0.45, *p = 0*.01) and the PPT-7 (*r*_xyz_ = 0.49, *p =* 0.01) even after adjusting for age. Peak knee flexor torque at 180°/s also exhibited significant age-adjusted partial correlation with customary gait (*r*_xyz_ = 0.50, *p* = 0.01) and the PPT-7 (*r*_xyz_ = 0.41, *p =* 0.03). A visual depiction of the zero-order bivariate strength-function relationships is featured in Figure 2.

## 4. Discussion

The main objective of this work was to examine the relationship of grip strength and knee extensor/flexor strength with common functional measures used in the identification and assessment of sarcopenia. Better understanding the relative value of upper and lower extremity strength testing for sarcopenia staging would provide additional guidance regarding the management of this geriatric syndrome. Understanding the strength-function relationships in this predominantly African American sample of older men was aided by the addition of lower extremity strength testing. Our results showed that peak knee extension torque at 60°/s was significantly correlated with all performance-based outcome measures, whereas peak grip force was only significantly correlated with fast gait speed. These findings suggest that lower extremity strength testing may serve as a better marker of muscle impairment for sarcopenia staging in some clinical and research settings.

### 4.1. Hand Grip Strength as a Proxy Measure for Lower Extremity Strength

The assessment of overall muscle strength and function is critical for accurate screening and staging of sarcopenia within older individuals. Hand grip dynamometry is used as a primary tool for evaluating global muscle strength to diagnosis and stage sarcopenia because of the test’s ease of use, reliability, and low cost [2,3,13,14]. In this study, we reported a poor association between hand grip strength and measures of knee extensor and flexor strength in a community-dwelling sample of older men. Our results are consistent with the low to fair relationships between hand grip strength and lower extremity strength observed in large population-based studies involving older cohorts [14,35,36]. Yeung et al. [37] also found only low to moderate agreement between hand grip strength and knee extension strength independently of age and health status in populations of both young and older patients. Moreover, Felicio et al. [38] found no correlation between lower extremity strength values obtained via isokinetic dynamometry and isometric grip strength obtained using a hand-held dynamometer. Given these findings, some investigators recommend that hand grip strength should not be equated with overall muscle strength [37,39]. In contrast, Bohannon et al. [40] found good correlations between grip strength and knee extension strength of the ipsilateral or contralateral sides (*r* = 0.77 to 0.81), which favor the use of grip strength as an acceptable measure for overall muscle strength. Differences in the observations of Bohannon et al. [40] from our study findings may be attributed to wide age range of their study participants (18–85 years of age), whereas the participants featured in this report were comparatively older (45–83 years of age). The concordance between grip strength and measures of lower extremity strength would be aided by a wide range of force or torque values. Therefore, attributes of the cohort or patient population should be carefully considered when determining the appropriate use of grip strength as a proxy measure of lower extremity or overall strength. Importantly, upper and lower extremity muscles differ in the frequency of their recruitment during daily living activities [40] and evidence suggests that lower extremity strength has a greater rate of age-related decline compared to upper extremity strength [41]. As a result, some investigators have noted that the use of grip strength as a proxy measure for lower extremity strength warrants caution.

### 4.2. The Association of Functional Performance with Hand Grip Strength and Lower Extremity Strength

Gait speed and other functional battery tests have been used as predictors of disability risk and health-related outcomes in older populations [10,14]. In addition, grip strength has been proposed as an alternative screening test to identify older adults with sarcopenia [42]. Therefore, efforts to better understand the association of muscle strength with gait speed and other performance-based assessment tools will further inform the clinical approach to sarcopenia screening and staging. The findings in this report suggest that knee extensor and flexor strength are more strongly associated with customary gait speed in comparison to hand grip strength. Our data concerning the relationship between customary gait speed and isokinetic knee extensor peak torque scaled for body weight is consistent with similar observations involving isometric knee extensor strength expressed as the unadjusted peak force [43]. In contrast, Fragala et al. [14] found that knee extensor strength only nominally improved on hand grip strength as a predictor of customary gait speed in adults ranging from 67 to 93 years old. Similarly, Chan et al. [36] reported that hand grip strength and knee extensor strength contributed similarly in a multivariate model of baseline health outcomes, such as recent hospitalization, customary gait speed, and health-related quality of life in a cohort of participants consisting of older adults in primary care over the age of 75. Knee extensor force may have a declining influence on gait speed and functional performance as older adults enter the 8th decade of life and beyond [17], which reflects how differences in sample characteristics may affect strength-function relationships. Notably, our older community-dwelling veteran participants (mean age, 62.5 ± 9.2) exhibited hand grip strength that was significantly associated with fast gait speed (*p =* 0.02), but not customary gait speed (*p =* 0.18). Our previous study of older adults with intrinsic muscle disease demonstrated that individuals may exhibit decrements in strength approaching 50% of normative values, yet still maintain walking speeds that exceed 1.0 m/s [44]. The redundancy in muscle group contributions to gait, and the role of ground reaction forces during stance phase, may obfuscate early muscle impairments when temporal gait parameters remain stable [25,44]. Consequently, more demanding functional tasks, such as the fast walking test [25], may show a stronger association with local and global measures of strength as shown in this report.

Customary gait speed remains a prominent feature of consensus-based screening procedures for sarcopenia [2,45]. However, other common sarcopenia outcome measures range from the timed sit-to-stand tests to multi-item performance-based functional tests. In this report, we further evaluated the functional status of the participants using the sit to stand test and the PPT-7. Our data suggested that lower extremity strength, but not hand grip strength, was significantly associated with the aforementioned functional tests. Specifically, knee extensor force was the sole strength variable significantly associated with the sit to stand test (*p* = 0.02). The relative lack of association of the sit to stand test with most of the strength measures was not wholly unexpected. Previous reports have cited the relative importance of tactile sensation, reaction time, and other measures of neuromuscular function to sit to stand test performance, rather than just lower extremity strength [46,47]. Moreover, Bohannon et al. [40] also noted that grip strength testing results do not provide a straightforward explanation for age-related declines in sit-to-stand performance due to the lack of common muscles needed to perform each task. Functional battery tests such as the PPT-7 have a larger testing burden than individual assessments such as the sit to stand test. Nevertheless, the PPT-7 is a clinically feasible test that has been validated for older adults and captures broad aspects of upright mobility. The participants in this study had lower extremity strength values that were moderately associated with PPT-7 performance (*r* = 0.45 to 0.57). The association of functional performance with lower extremity strength – specifically, knee flexor strength – was significant in this study even when adjusting for participant age. Using a similar methodological approach to this study, Martien et al. [16] also evaluated the relationship of PPT-7 scores in older adults with hand grip and knee extensor strength values scaled to body weight. In cohorts involving both nursing home and assisted living residents, multivariate models accepted the variable for knee extensor strength and omitted grip strength as a significant predictor of PPT-7 performance (*R*^2^ = 0.35 to 0.37, *p* < 0.05). The data from this report featuring a sample of community-dwelling older men, along with a cohort of institutionalized older adults reported by Martien et al. [16], provide additional insights concerning the primacy of lower extremity strength for functional performance when peak torque or force values are adjusted for body size [48].

### 4.3. Potential Uses of Lower Extremity Strength Assessment in the Management of Sarcopenia

There is strong evidence in the literature that demonstrates sarcopenia to be a reversible cause of disability, and that people in early stages of sarcopenia would potentially benefit from intervention [3]. Therefore, the use of valid biomarkers and endpoint measures of sarcopenia are critical to the pursuit of both clinical trials and general case management [49,50]. In considering the EWGSOP staging model [2], one may transition from a sarcopenic state in one of three ways: (1) increasing muscle mass, (2) improving functional performance, or (3) generating higher levels of muscle force [51]. Ostensibly, many individuals engaging in strength training programs may transition from the “sarcopenia” stage to the “presarcopenia” stage by gaining strength while still remaining below the criterion for normal muscle mass. Tieland et al. [52] found that grip strength did not reflect the changes in general strength following the implementation of an exercise intervention. While no significant changes were detected when comparing pre- and post-intervention measurements of grip strength in the exercise group, leg extension strength and functional status increased significantly following a 24-week whole body progressive resistance exercise program. Increasingly, it is becoming apparent that changes in lower extremity strength and function do not consistently mirror corresponding changes in hand grip strength [39,41,52]. Hand grip dynamometry remains an important tool to obtain surrogate measures of strength, and a useful predictor of patient outcomes in population-based studies. However, investigators should be mindful of the inherent limitations associated with the use of surrogate endpoint measures. Hand grip strength may help to identify sarcopenia, and yet constitute a surrogate endpoint measure that is not adequately responsive to exercise interventions for sarcopenia [49]. While lower extremity strength measures were significantly associated with physical performance in this study, it is important to note that any isolated measure of strength or function could under-represent the larger deficits associated with geriatric syndromes such as sarcopenia or frailty. Although lower extremity and hand grip strength have been shown to be comparable regarding their relationship with gait speed and hospitalization risk in large population-based studies [14,53], the findings from this report and other investigators [39,52] suggest that case management and longitudinal clinical studies would be enhanced by a more broad assessment of muscle strength.

The current study has limitations which affect the interpretation and generalizability of the results. The population tested included only 30 participants who were recruited and enrolled at a Federal hospital. The participants were community-dwelling older adults, which represents a differing strength-function profile from institutionalized adults or those with significant comorbid conditions. Additionally, this predominantly African American Veteran sample was recruited from a major urban city and may vary considerably from individuals recruited from rural settings or small community hospitals. The study sample was limited to male participants given its setting in a VA medical facility. Consequently, the reported strength-function relationships may vary with the inclusion of older women. This limitation is important to note since it has been shown that men and women demonstrate different trajectories in muscle deterioration associated with aging [54]. However, the general observation that hand grip strength may under-represent the construct of global strength has been noted in cohort studies featuring female participants [37]. Additionally, the present study incorporated the PPT-7 as the primary assessment of functional status. While the PPT-7 is well validated for use with older adults [31], the Short Physical Performance Battery (SPPB) [11] is commonly used in sarcopenia clinical trials. Examining the relationship of SPPB scores with upper and lower extremity strength values may yield results that differ from the findings of this report.

## 5. Conclusions

Our results demonstrated significant associations between most measures of lower extremity strength and functional performance, whereas limited associations were observed with hand grip strength as the primary outcome measure. We found that hand grip strength was not a suitable proxy measure of lower extremity strength. Our findings affirm the observation that lower extremity strength testing reflects functional performance in older community-dwelling adults, and that lower extremity strength measurement in the form of peak knee extensor and flexor torque at 60°/s would provide additional value to the sarcopenia staging process in both clinical and research settings.

## Figures and Tables

**Table 1:** Study participants.

Study Participant Demographics	
Age (years)	62.5 ± 9.2
Body mass index	26.3 ± 3.8
Height (cm)	177.1 ± 6.8
Weight (kg)	82.5 ± 13.1
Lean body mass (kg/m^2^)	8.57 ± 1.12
Total adiposity (body fat, %)	27.8 ± 7.4
Racial/ethnic group	
African American	24
Caucasian	6

Data are expressed as mean values ± SD, except whre indicated. Body composition was estimated using dual-energy X-ray absorptiometry. Fat-free mass was estimated in the arms and legs (aLM) and scaled to body size (aLM/ht^2^). Body mass index was calculated as weight/height (kg/m^2^).

**Table 2:** Physical performance measures.

**Muscle Strength**		
	**Mean**	**SD**
Peak Grip Force (0°/s)	0.49	±0.98
Peak Knee Extensor Torque (60°/s)	0.58	±0.16
Peak Knee Extensor Torque (180°/s)	0.37	±0.12
Peak Knee Flexor Torque (60°/s)	0.26	±0.09
Peak Knee Flexor Torque (180°/s)	0.18	±0.07
**Functional Performance**		
	**Mean**	**SD**
Customary Gait Speed (m/s)	1.23	±0.34
Fast Gait Speed (m/s)	1.62	±0.41
Sit to Stand (s)	11.75	±4.01
PPT-7	21.2	±3.1

Notes: PPT-7, 7-item Physical Performance Test; all strength values are scaled to body weight.

**Table 3:** Strength-function relationships.

		Customary Gait Speed	Fast Gait Speed	Sit to Stand	PPT-7
Peak Grip Force (0°/s)	*r*	0.25	0.42	−0.32	0.33
	*p*-value	0.175	**0.021**	0.084	0.079
Peak Knee Extensor Torque (60°/s)	*r*	0.47	0.46	−0.43	0.45
	*p*-value	**0.009**	**0.010**	**0.017**	**0.013**
Peak Knee Extensor Torque (180°/s)	*r*	0.45	0.31	−0.27	0.29
	*p*-value	**0.013**	0.101	0.146	0.124
Peak Knee Flexor Torque (60°/s)	*r*	0.41	0.52	−0.35	0.55
	*p*-value	**0.026**	**0.004**	0.067	**0.002**
Peak Knee Flexor Torque (180°/s)	*r*	0.59	0.41	−0.37	0.57
	*p*-value	**0.001**	**0.028**	0.051	**0.001**

Notes: PPT-7, 7-item Physical Performance Test; *r*, Pearson correlation (*p* < 0.05 in bold).
